# Egg shape changes at the theropod–bird transition, and a morphometric study of amniote eggs

**DOI:** 10.1098/rsos.140311

**Published:** 2014-11-05

**Authors:** D. Charles Deeming, Marcello Ruta

**Affiliations:** School of Life Sciences, Joseph Banks Laboratories, University of Lincoln, Green Lane, Lincoln LN6 7DL, UK

**Keywords:** birds, Cenozoic, eggshell outline, mesozoic, morphospace, theropods

## Abstract

The eggs of amniotes exhibit a remarkable variety of shapes, from spherical to elongate and from symmetrical to asymmetrical. We examine eggshell geometry in a diverse sample of fossil and living amniotes using geometric morphometrics and linear measurements. Our goal is to quantify patterns of morphospace occupation and shape variation in the eggs of recent through to Mesozoic birds (neornithe plus non-neornithe avialans), as well as in eggs attributed to non-avialan theropods. In most amniotes, eggs show significant deviation from sphericity, but departure from symmetry around the equatorial axis is mostly confined to theropods and birds. Mesozoic bird eggs differ significantly from extant bird eggs, but extinct Cenozoic bird eggs do not. This suggests that the range of egg shapes in extant birds had already been attained in the Cenozoic. We conclude with a discussion of possible biological factors imparting variation to egg shapes during their formation in the oviduct.

## 2. Introduction

The origin of the amniotic egg is one of the key adaptations underpinning vertebrate terrestrialization. Extant amniote eggs vary considerably in shape and size [1]. In non-avian amniotes, two extremes are the almost spherical eggs of marine turtles and the highly elongate eggs of some snakes [2]. Both types are ellipsoidal, i.e. symmetrical about their long (polar) and short (equatorial) axes. By contrast, avian eggs range from nearly spherical (e.g. some owls [3]) to markedly asymmetrical (ovoidal) about their short axes (e.g. some shorebirds [4]), and many intermediate shapes occur in between [5].

This work investigates changes in eggshell geometry across the transition from non-avialan theropods (hereafter, theropods for simplicity) to fossil (both Cenozoic and Mesozoic) and living avialans (hereafter, birds for simplicity), and includes a small sample of eggs from other amniote groups for general comparisons. Taxonomic attributions of fossil eggs often rest on scanty evidence. This issue is particularly acute in the case of theropod and Mesozoic bird eggs. Our study provides a frame of reference for determining whether egg shape is a reliable indicator of taxonomic groupings, and we hope that future discoveries of unequivocally associated fossil eggs and skeletons will help refine the general conclusions of the present investigation.

Our goals are: (i) to examine the distribution in morphospace of egg types from a diverse range of amniote groups, both extinct and extant; (ii) to evaluate similarities/dissimilarities among eggs belonging to Mesozoic, extinct Cenozoic and extant birds, and to compare each of those categories to eggs attributed to theropods.

Here, we apply the term ‘bird’ to the greatest majority of Mesozoic stem-group birds (non-neornithe avialans; note: there are some unequivocal crown-group birds occurring just before the Cretaceous/Paleogene boundary [6]), as well as to extinct Cenozoic and extant crown-group birds (neornithe avialans). We apply the term ‘theropod’ to the grade group of pre-avialan bipedal saurischian dinosaurs.

## 3. Material and methods

**Figure 1.**
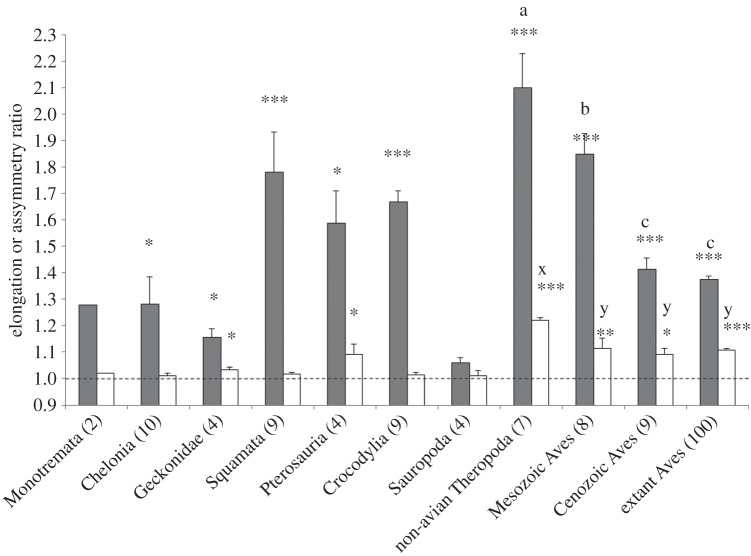
Mean plus standard error values for elongation ratio (ER, grey columns) and asymmetry ratio (AR, white columns) in 11 amniote groups. Asterisks above a column indicate that the mean is significantly different from 1 (*p<0.05; ***p< 0.001). For non-avian theropods and Aves, columns differing in letters x and y or a, b and c exhibit significant differences between means at *p*<0.05.

Previous works have characterized egg shape in terms of either linear dimensions or curve-fitting equations [5,7,8]. However, egg outlines may differ even when their linear dimensions are identical. Here, we quantify egg shape both with geometric morphometric methods applied to two-dimensional landmarks along the egg outlines [9], and with traditional morphometric methods applied to linear dimensions. Egg photographs from 11 amniote groups were collated from literature and online sources (figure 1; electronic supplementary material, table S1). Forty equally spaced landmarks were digitized along the right-hand side of each egg outline using tpsDig2 v. 2.17 [10]. The landmark coordinates were used to carry out semi-landmark and eigenshape analyses [11–15], respectively, in MorphoJ v. 1.05f [16] and PAST v. 2.17c [17]. The semi-landmark analysis [11–13] is a principal component analysis of the variance–covariance matrix of Procrustes-fitted landmark coordinates, i.e. coordinates obtained after removal of scale, translation and rotation. The eigenshape analysis [14,15] is a principal component analysis of the variance–covariance matrix of turning angle increments along the egg outlines. For each analysis, we built a two-dimensional morphospace plot based on the first two shape axes (labelled as PC and ES for the semi-landmark and eigenshape analyses, respectively).

The scores (coordinates) of egg types along those axes were used to quantify group separation in morphospace through a non-parametric multivariate analysis of variance (npMANOVA) [18] and a non-parametric analysis of similarity (ANOSIM) [19] in PAST, with Bonferroni correction of *p*-values for multiple comparisons. npMANOVA tests whether the distributions of scores for various egg groups differ (H_0_: different groups have similar variances [17,18]; the significance of the test is evaluated through permutations of taxon assignments to the groups: briefly, taxa are randomly assigned to the selected groups in proportion to the number of species in each group; this random assignment is repeated numerous times, and each time the test statistic is calculated; the observed statistic is then compared with the distribution of statistic values generated from the permutations). ANOSIM tests whether the rank-converted distances among egg types within the original groups to which they belong differ from the rank-converted distances among egg types after the latter are assigned to groups at random, and in identical proportion to the numbers that constitute the original groups (H_0_: ranked dissimilarities within groups have equal median and range [17,19]; as in the case of npMANOVA, the taxa are assigned at random to the groups, and a test statistic is calculated at the end of each random permutation of taxa; the significance of the statistic for the original groups is assessed via comparison with the distribution of the statistic values from the random permutations).

The maximum length (*L*) and breadth (*B*) of each egg were recorded in ImageJ v. 1.47 [20] and used to calculate the elongation ratio (ER=*L*/*B*). The distance *D* from the lower vertex of the egg to the point where the polar axis intersects the equatorial axis was used to calculate the asymmetry ratio (AR=*D*/*L*). Departures of ER and AR from 1 (the values of a sphere) were assessed with one-sample *t*-tests and ANOVA in Minitab v. 15.

## 4. Results

In regard to egg elongation, significant ER departures from 1 characterize all groups except monotremes (small sample size) and sauropods (near spherical eggs) (figure 1; *F*_10,154_=20.1, *p*<0.001). Theropods exhibit the most elongate eggs, followed by those of Mesozoic birds and squamates. ER was greatest in theropods but smallest in extant birds, with significant differences between these groups (*F*_3,120_=70.98, *p*<0.001). All pairwise comparisons between groups were significant, except in the case of extinct Cenozoic versus extant birds. In regard to egg asymmetry, significant AR departures from 1 characterize hard-shelled geckos, pterosaurs, theropods and birds, whereas other groups exhibit symmetrical or near symmetrical eggs (figure 1; *F*_10,154_=7.77, *p*<0.001). In theropods, there was a significant effect of taxon on AR (*F*_3,120_=5.29, *p*=0.002). Theropod eggs are significantly more asymmetrical than all other bird egg types, and the latter do not differ significantly from each other.

**Figure 2.**
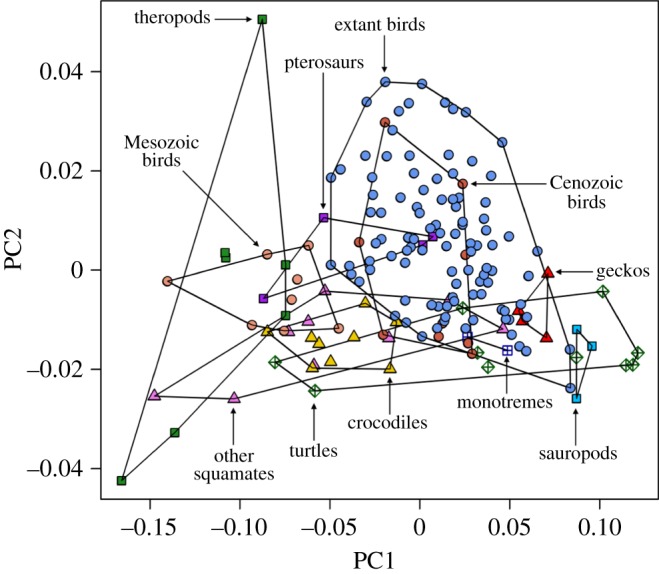
Morphospace plot based on the semi-landmark analysis of egg types, with convex hulls delimiting the 11 groups in the dataset. On PC1, increasingly elongate (respectively, spherical) eggs are associated with increasingly negative (respectively, positive) scores. On PC2, increasingly symmetrical (respectively, asymmetrical) eggs are associated with increasingly negative (respectively, positive) scores.

**Figure 3.**
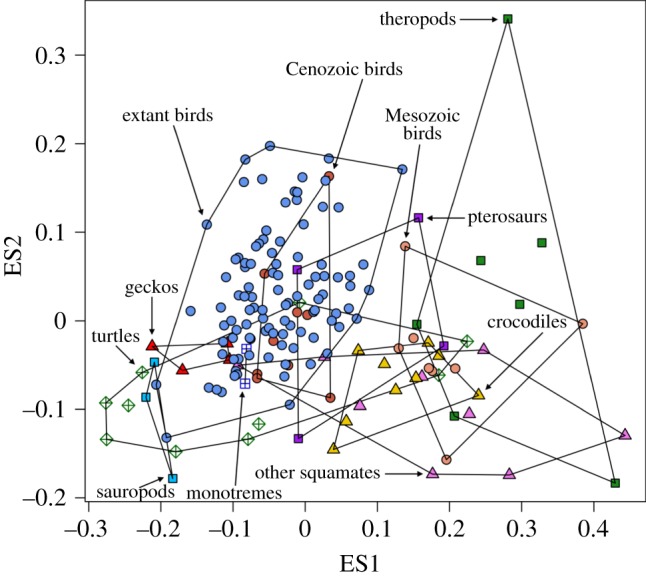
Morphospace plot based on the eigenshape analysis of egg types, with convex hulls delimiting the 11 groups in the dataset. On ES1, increasingly elongate (respectively, spherical) eggs are associated with increasingly positive (respectively, negative) scores. On ES2, increasingly symmetrical (respectively, asymmetrical) eggs are associated with increasingly negative (respectively, positive) scores.

The first three shape axes summarize 89.48, 7.96 and 1.27% of the total variance in the semi-landmark analysis, and 41.69, 17.52 and 9.96% of the total variance in the eigenshape analysis. The two-dimensional morphospace plots from the semi-landmark and eigenshape analyses are very similar in terms of relative positions of egg types (the eigenshape plot is merely flipped horizontally relative to the semi-landmark plot; figures 2 and 3). In the following, therefore, we focus on the PC plot only to describe patterns of morphospace occupation (figure 2). On PC1, increasingly elongate (respectively, spherical) eggs are associated with increasingly negative (respectively, positive) scores. On PC2, increasingly symmetrical (respectively, asymmetrical) eggs are associated with increasingly negative (respectively, positive) scores.

Concerning the distribution of egg shapes in morphospace, the following main features are noteworthy. First, the ensemble of non-avian amniote eggs tend to be concentrated in the lower half of the plot, implying limited variation across the range of symmetrical–asymmetrical shapes but extensive variation in the range of spherical–elongate shapes. Second, theropod eggs display greater variation in their asymmetry than in their sphericity and are generally elongate. Third, Mesozoic bird eggs are slightly more elongate than the greatest majority of fossil Cenozoic and extant bird eggs, but fall within the range of asymmetry variation of the latter. Fourth, extant bird eggs vary considerably in their degree of asymmetry, but do not reach the two theropod extremes represented by the highly symmetrical ootaxon *Macroelongatoolithus* at the bottom of the plot and the highly asymmetrical *Troodon* eggs at the top. Fifth, extant bird eggs plot out mainly in the right half of the PC plane, ranging in shape from mid-elongate to nearly spherical.

As regards non-avian amniote eggs, most of them show varying degrees of elongation but similar degrees of asymmetry. Squamate and turtle eggs exhibit remarkable ER variation (e.g. rigid-shelled gecko eggs and marine turtle eggs are more spherical than those of other squamates and turtles), while crocodile eggs form a compact cluster in morphospace (figure 2). Cenozoic bird eggs occur within the range of extant bird eggs, but Mesozoic bird eggs are distinctly separate and morphologically intermediate between those of theropods and those of other birds (figure 2). By contrast, two fossil crocodile eggs and a single fossil turtle egg in our sample occur well within the range of shape variation exhibited by their extant counterparts. Global tests of group separation in morphospace (for both semi-landmark and eigenshape analyses) produce significant results (*p*=0.0001) in both npMANOVA and ANOSIM. Significant pairwise comparisons are highlighted in grey cells in table 1. In the two morphometric analyses, both theropod eggs and Mesozoic bird eggs differ significantly from those of fossil Cenozoic birds and from those of extant birds. However, theropod and Mesozoic bird eggs are not significantly different.

**Table 1.** Results of npMANOVA and ANOSIM tests conducted on PC1–2 and ES1–2 scores. For each pairwise comparison, the values of the *F* (npMANOVA) and *R* (ANOSIM) statistics are reported above the diagonal. The level of significance for each comparison is reported below the diagonal. Grey cells show significant comparisons.

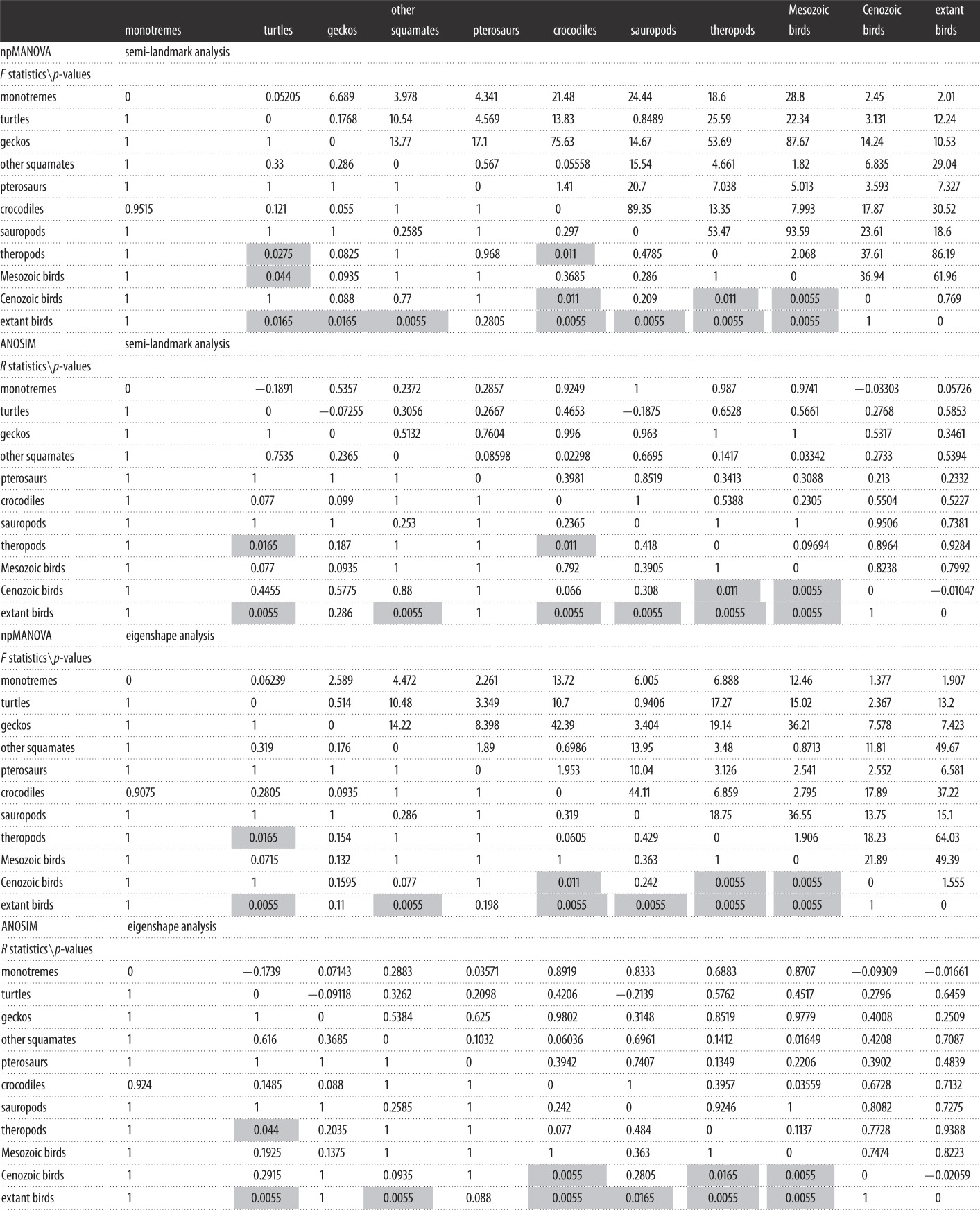

**Figure 4.**
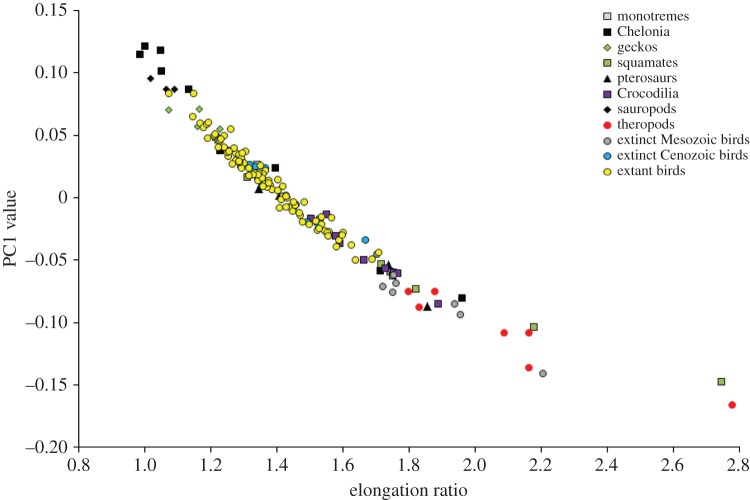
Relationship between elongation ratio values (maximum length divided by maximum breadth) and PC1 scores. On PC1, increasingly elongate (respectively, spherical) eggs are associated with increasingly negative (respectively, positive) scores.

**Figure 5.**
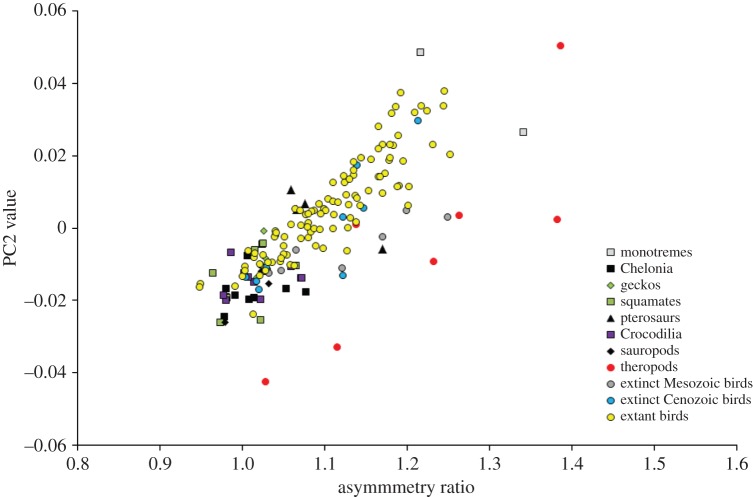
Relationship between asymmetry ratio values (distance from lowest vertex of egg to point were polar and equatorial axes cross divided by maximum egg breadth) and PC2 scores. On PC2, increasingly symmetrical (respectively, asymmetrical) eggs are associated with increasingly negative (respectively, positive) scores.

PC1 scores exhibit a significant and strong negative correlation with ER values, whereas PC2 scores do not (PC1 versus ER: *r*_s_=−0.991, *p*<0.001, figure 4; PC2 versus ER: *r*_s_=0.072, *p*=0.359). Both PC1 and PC2 scores are significantly correlated with AR values, with a weak negative correlation for PC1 and a strong positive correlation for PC2 (PC1 versus AR: *r*_s_=−0.274, *p*<0.001; PC2 versus AR: *r*_s_=0.831, *p*<0.001, figure 5).

## 5. Discussion

ER separates amniote eggs more readily than AR. Most extant amniotes lay eggs that are asymmetrical to some degree. By contrast, theropods exhibit variously elongate symmetrical eggs, one extreme being the theropod ootaxon *Macroelongatoolithus* [21] with an ER of 2.778. In terms of both ER and AR, Mesozoic bird eggs are morphologically intermediate between extant bird eggs and theropod eggs. However, despite a flurry of Mesozoic bird discoveries (electronic supplementary material, table S1), fossil avian eggs remain rare, and in the absence of associated skeletons their attribution to either theropods or birds is problematic. As an additional complication, eggshell characteristics are not necessarily diagnostic for theropod or avian eggs [22]. For instance, our sample includes two eggs ascribed to theropods [23,24], but these appear to be morphologically more consistent with Mesozoic bird eggs.

From a biological perspective, it is almost axiomatic to assume that the different egg shapes exhibited by birds, both past and present, might be associated with different nesting behaviours and/or incubation modes. Surprisingly, however, hardly any research has been carried out on this topic. In addition, fossil data are insufficient to draw firm conclusions. In very few cases, however, separate evidence suggests that some Mesozoic birds at least differed from extant birds. For instance, the eggshells of *Gobipipus* are more porous than expected from their predicted size, suggesting a mode of incubation similar to that of crocodiles [25]. An interesting implication of our results is that extinct Cenozoic bird eggs fit squarely within the range of shape variation of extant crown-group bird eggs, suggesting that this shape variation had been attained in the Cenozoic. The dearth of extinct Cenozoic and Mesozoic bird eggs prevents us from providing a more accurate picture of the chronology of significant shifts in shape change in the transition from non-neornithe to neornithe avialans.

Turning now to reproductive biology, we speculate on possible biological factors that might be responsible for the variety of egg shapes observed in amniotes, and we conclude our narrative with some final thoughts on the reproductive biology of theropods. Upon ovulation, all yolk-rich vertebrate ova are spherical, so alternative shapes are conferred during the accumulation of any albumen and deposition of the eggshell in the oviduct. The deposition of a thick calcareous shell does not impair the ability of some amniotes to produce spherical eggs (e.g. sauropods and some turtles), though in other groups (e.g. crocodiles and other turtles) eggs are more elongate. Presumably, given the physical restraints of a long oviduct, secretion of large amounts of albumen implies that eggs become more elongate while albumen is deposited at either extremity of the egg, with very little amount present around the spherical yolk, as in extant bird eggs [26] and crocodiles [27].

However, albumen is absent in extant squamate eggs and analysis of freshly laid lizard eggs (D. C. Deeming 2014, personal observation) suggests that their elongation is associated with accumulation of sub-embryonic fluid. Produced during early embryogenesis, and presumably derived from the oviduct, the sub-embryonic fluid expands the volume of the yolk. Given the constraint of the oviduct lumen, volume increase can only be attained when the yolk is squeezed into an elongate shape before shell deposition. This probably applies to a lesser extent to the elongate, hard-shelled crocodile eggs. Such eggs also contain well-developed embryos at a developmental stage equivalent to that of bird embryos when the production of sub-embryonic fluid results in yolk expansion *in ovo* [28].

If embryo physiology is indeed a key factor in the production of elongate eggs, then it would be interesting to investigate development in other amniotes with elongate eggs. As an example, embryological studies of turtles to date have focused primarily on species laying spherical eggs [29,30], in which embryos are at a relatively early developmental stage. However, we do not know whether, at oviposition, the embryos of turtles that lay elongate eggs are at a later developmental stage.

How did theropods produce extremely elongate eggs? We speculate that the ovulated ova could not have been much larger than the oviduct lumen. As the incubation period is a function of egg size and yolk content, a very large and elongate egg presumably did not have a small yolk and did not include a large amount of albumen, for two reasons. Firstly, the diameter of the oviduct would be limited. Therefore, given that the ovum is not forced into the oviduct after ovulation, there would be no mechanism to squeeze a large diameter ovum into the oviduct, implying that the ovum was approximately the same size as the oviduct's diameter. Second, incubation period correlates positively with energy content [31]. This would have restricted the ability of an embryo to incorporate all of the contents (particularly its ability to transfer water from the albumen to the ovum) during a short incubation period. Absorption of water from the oviduct prior to shell deposition implies that the incubation duration could be shortened. We hypothesize that theropod eggs lacked albumen and that water required for development was absorbed from the oviduct by the developing embryo, thus causing the ovum to expand greatly. During this process, constriction by the oviduct would have forced the ovum into an ellipsoid shape before the shell was deposited. Therefore, we posit that, in stark contrast to extant birds [32], some theropod embryos may have been at an advanced developmental stage at oviposition.

As a concluding remark, it is unclear why egg asymmetry evolved in theropods. In some extant birds, however, this asymmetry may be related to the need for adults to incubate clutches ([33], but see [34]), although additional data are sorely needed. We are now in the process of investigating the highly variable egg composition in birds as a possible determinant of egg shape.

## Supplementary Material

Species list and data

## Data accessibility

Data available from the Dryad Digital Repository: http://dx.doi.org/10.5061/dryad.s9227.
