# Supplementary material for: Egg shape changes at the theropod–bird transition, and a morphometric study of amniote eggs
Source: R Soc Open Sci. 2014 Nov 5;1(3):140311. doi: 10.1098/rsos.140311 (PMC4448846; doi:10.1098/rsos.140311)
Supplement: Species list and data [file rsos140311supp1.pdf]

Deeming & Ruta **Egg ratio data used in analyses**

Class	Order	Geological Era	Genus	Species	Elongation ratio (ER)	Asymmetry ratio (AR)	Image reference
Mammalia	Monotremata	Modern	*Tachyglossus*	*aculeatus*	1.341	1.040	1
Mammalia	Monotremata	Modern	*Ornithorhynchus*	*anatinus*	1.216	1.003	2
Reptilia	Chelonia	Modern	*Chelydra*	*serpentina*	1.000	0.980	3
Reptilia	Chelonia	Modern	*Chelonoidis*	*carbonaria*	1.228	1.008	4
Reptilia	Chelonia	Modern	*Tesudo*	*marginata*	1.050	1.024	5
Reptilia	Chelonia	Modern	*Chelonoidis*	*chilensis*	1.288	1.053	6
Reptilia	Chelonia	Modern	*Geoemyda*	*japonica*	1.961	0.991	7
Reptilia	Chelonia	Modern	*Pseudemydura*	*umbrina*	1.712	0.978	8
Reptilia	Chelonia	Modern	*Terrapene*	*ornata*	1.396	1.006	9
Reptilia	Chelonia	Modern	*Dermochelys*	*coriacea*	0.986	1.014	10
Reptilia	Chelonia	Modern	*Caretta*	*caretta*	1.049	0.980	11
Reptilia	Chelonia	Cretaceous	?	-	1.132	1.077	12
Reptilia	Squamata (Gecko)	Modern	*Hemidactylus*	*turcicus*	1.229	1.028	13
Reptilia	Squamata (Gecko)	Modern	*Hemidactylus*	*frenatus*	1.074	1.017	14
Reptilia	Squamata (Gecko)	Modern	*Phelsuma*	*bobonica*	1.167	1.026	15
Reptilia	Squamata (Gecko)	Modern	*Phelsuma*	*standii*	1.161	1.064	16
Reptilia	Squamata	Modern	*Eublepharis*	*macularius*	1.742	0.981	4
Reptilia	Squamata	Modern	*Pogona*	*vitticeps*	1.530	1.071	4
Reptilia	Squamata	Modern	*Lacerta*	*schreiberi*	1.224	1.030	17
Reptilia	Squamata	Modern	*Iguana*	*iguana*	1.310	1.015	18
Reptilia	Squamata	Modern	*Pantherophis*	*guttatus*	1.715	1.025	19
Reptilia	Squamata	Modern	*Pantherophis*	*guttatus*	2.178	0.973	20
Reptilia	Squamata	Modern	*Pseudonaja*	*textilis*	2.746	1.022	21
Reptilia	Squamata	Modern	*Python*	*regius*	1.750	1.065	22
Reptilia	Squamata	Modern	*Pseudonaja*	*textilis*	1.819	0.964	23
Pterosauria	-	Cretaceous	*Darwinopterus*	-	1.408	1.066	24
Pterosaur	-	Cretaceous	*Hamipterus*	*tianshanensis*	1.856	1.170	25
Pterosaur	-	Cretaceous	*Hamipterus*	*tianshanensis*	1.345	1.076	25
Pterosauria	-	Cretaceous	*?*	-	1.738	1.059	26
Reptilia	Crocodylia	Modern	*Alligator*	*mississippiensis*	1.729	1.014	4
Reptilia	Crocodylia	Modern	*Caiman*	*crocodilus*	1.753	1.022	27
Reptilia	Crocodylia	Modern	*Crocodylus*	*porosus*	1.502	0.980	28
Reptilia	Crocodylia	Modern	*Crocodylus*	*rhombifer*	1.889	1.002	29
Reptilia	Crocodylia	Modern	*Crocodylus*	*niloticus*	1.664	0.977	30
Reptilia	Crocodylia	Modern	*Tomistoma*	*schlegelii*	1.592	1.007	31

Reptilia	Crocodylia	Modern	*Gavialis*	*gangeticus*	1.551	1.059	32
Reptilia	Crocodylia	Cretaceous	?	-	1.578	0.986	33
Reptilia	Crocodylia	Eocene	?	-	1.766	1.072	34
Dinosauria	Sauropoda	Cretaceous	?	-	1.018	1.032	35
Dinosauria	Sauropoda	Cretaceous	?	-	1.065	1.022	36
Dinosauria	Sauropoda	Cretaceous	?	-	1.092	0.979	37
Dinosauria	Theropoda	Cretaceous	*Continuoloothis*	sp.	2.163	1.115	38
Dinosauria	Theropoda	Cretaceous	*Macroelongatoolithus*	sp.	2.778	1.028	39
Dinosauria	Theropoda	Cretaceous	*Protoceratopsidovum*	*sincerum*	2.163	1.382	40
Dinosauria	Theropoda	Cretaceous	*Elongatoolithis*	sp.	2.088	1.263	41
Dinosauria	Theropoda	Cretaceous	?	-	1.799	1.138	42
Dinosauria	Theropoda	Cretaceous	*Sankofa*	*pyrenaica*	1.878	1.231	43
Dinosauria	Theropoda	Cretaceous	*Troodon*	*formosus*	1.831	1.386	44
Mesozoic bird	-	Cretaceous	?	-	1.703	1.047	45
Mesozoic bird	-	Cretaceous	?	-	1.750	1.032	46
Mesozoic bird	-	Cretaceous	*Neuquenornis*	sp.	1.753	1.199	47
Mesozoic bird	-	Cretaceous	*Styloothis*	sp.	2.206	1.170	48
Mesozoic bird	-	Cretaceous	*Gobioolithis*	*major*	1.721	1.065	48
Mesozoic bird	-	Cretaceous	*Gobioolithis*	*minor*	1.954	1.121	48
Mesozoic bird	-	Cretaceous	*Gobipipus*	sp.	1.938	1.249	49
Mesozoic bird	-	Cretaceous	*Gobioolithus*	*minor*	1.760	1.040	40
Cenozoic bird	Sphenisciformes		*Eudyptula*	*minor*	1.335	1.122	51
Cenozoic bird	Procellariiformes	Pleistocene	Albatross	sp.	1.523	1.122	52
Cenozoic bird	Pelecaniformes	Pleistocene	*Pelecanus*	sp.	1.668	1.147	52
Cenozoic bird	Galliformmes	Pliocene	*Francolinus*	sp.	1.365	1.139	53
Cenozoic bird	Gruiiformes	Pliocene	*Badistornis*	*aramus*	1.297	1.020	54
Cenozoic bird	Charadriiformes	Holocene	*Pinguinus*	*impennis*	1.492	1.213	55
Cenozoic bird	Struthioniformes	Holocene	*Aepyornis*	*maximus*	1.319	1.017	56
Cenozoic bird	Struthioniformes	Holocene	*Anomalopteryx*	*didformis*	1.353	1.029	57
Cenozoic bird	Struthioniformes	Holocene	*Euryapteryx*	*gravis*	1.374	1.004	57
Extant bird	Struthioniformes	Modern	*Struthio*	*camelus*	1.211	1.058	4
Extant bird	Struthioniformes	Modern	*Dromaius*	*novaehollandiae*	1.471	1.046	58
Extant bird	Struthioniformes	Modern	*Casuarius*	*casuarius*	1.381	1.054	4
Extant bird	Struthioniformes	Modern	*Rhea*	*americana*	1.418	1.028	4
Extant bird	Apterygiformes	Modern	*Apteryx*	*owenii*	1.600	1.138	59
Extant bird	Apterygiformes	Modern	*Apteryx*	*australis*	1.536	1.201	60
Extant bird	Sphenisciformes	Modern	*Aptenodytes*	*forsteri*	1.545	1.178	61
Extant bird	Sphenisciformes	Modern	*Aptenodytes*	*patagonicus*	1.467	1.209	62
Extant bird	Sphenisciformes	Modern	*Pygoscelis*	*adeliae*	1.224	1.014	63

Extant bird	Sphenisciformes	Modern	*Pygoscelis*	*papua*	1.169	1.036	64
Extant bird	Anseriformes	Modern	*Anas*	*clypeata*	1.444	1.128	65
Extant bird	Anseriformes	Modern	*Anas*	*platyrhynchos*	1.450	1.080	65
Extant bird	Anseriformes	Modern	*Netta*	*rufina*	1.367	1.063	65
Extant bird	Anseriformes	Modern	*Tadorna*	*ferruginea*	1.385	1.050	65
Extant bird	Anseriformes	Modern	*Tadorna*	*tadorna*	1.344	1.025	65
Extant bird	Anseriformes	Modern	*Anser*	*anser*	1.637	1.059	65
Extant bird	Anseriformes	Modern	*Branta*	*canadensis*	1.444	1.003	66
Extant bird	Anseriformes	Modern	*Cygnus*	*olor*	1.627	1.040	65
Extant bird	Phoenicopteriformes	Modern	*Phoenicopterus*	*ruber roseus*	1.559	1.190	4
Extant bird	Galliformmes	Modern	*Gallus*	*gallus*	1.316	1.098	4
Extant bird	Galliformmes	Modern	*Alectura*	*rufa*	1.366	1.123	4
Extant bird	Galliformmes	Modern	*Perdix*	*perdix*	1.370	1.137	4
Extant bird	Galliformmes	Modern	*Phasianus*	*colchicus*	1.240	1.092	65
Extant bird	Gaviiformes	Modern	*Gavia*	*immer*	1.566	1.131	71
Extant bird	Podicipediformes	Modern	*Podiceps*	*nigricollis*	1.403	1.087	65
Extant bird	Pelecaniformes	Modern	*Phalacrocorax*	*carbo*	1.581	1.071	65
Extant bird	Ciconiiformes	Modern	*Ciconia*	*ciconia*	1.589	1.051	65
Extant bird	Ciconiiformes	Modern	*Egretta*	*garzetta*	1.275	1.022	65
Extant bird	Ciconiiformes	Modern	*Ardea*	*herodias*	1.358	1.007	66
Extant bird	Ciconiiformes	Modern	*Ardea*	*cincera*	1.324	1.071	65
Extant bird	Ciconiiformes	Modern	*Nycticorax*	*nycticorax*	1.418	1.000	65
Extant bird	Falconiformes	Modern	*Buteo*	*regalis*	1.306	1.104	65
Extant bird	Falconiformes	Modern	*Buteo*	*lineatus*	1.260	0.949	66
Extant bird	Falconiformes	Modern	*Buteo*	*swainsoni*	1.212	1.021	66
Extant bird	Falconiformes	Modern	*Buteo*	*jamaicensis*	1.179	1.015	66
Extant bird	Falconiformes	Modern	*Circus*	*aeruginosus*	1.293	1.050	65
Extant bird	Falconiformes	Modern	*Gyps*	*fulvus*	1.290	1.003	65
Extant bird	Falconiformes	Modern	*Falco*	*tinniculus*	1.254	1.039	65
Extant bird	Falconiformes	Modern	*Falco*	*peregrinus*	1.256	1.080	66
Extant bird	Gruiiformes	Modern	*Grus*	*canadensis*	1.689	1.195	66
Extant bird	Gruiiformes	Modern	*Rallus*	*limicola*	1.363	1.110	66
Extant bird	Gruiiformes	Modern	*Gallinula*	*chloropus*	1.553	1.128	65
Extant bird	Gruiiformes	Modern	*Fulica*	*atra*	1.459	1.084	65
Extant bird	Gruiiformes	Modern	*Chlamydotis*	*undulata*	1.191	1.021	4
Extant bird	Charadriiformes	Modern	*Gallingo*	*gallingo*	1.427	1.192	65
Extant bird	Charadriiformes	Modern	*Himatopus*	*himatopus*	1.455	1.186	65
Extant bird	Charadriiformes	Modern	*Recurirostra*	*avosetta*	1.337	1.224	65
Extant bird	Charadriiformes	Modern	*Numenius*	*americanus*	1.478	1.245	66

Extant bird	Charadriiformes	Modern	*Limosa*	*limosa*	1.535	1.165	65
Extant bird	Charadriiformes	Modern	*Charadrius*	*vociferus*	1.344	1.216	66
Extant bird	Charadriiformes	Modern	*Charadrius*	*semipalmatus*	1.229	1.188	66
Extant bird	Charadriiformes	Modern	*Vanellus*	*vanellus*	1.351	1.181	66
Extant bird	Charadriiformes	Modern	*Larus*	*argentatus*	1.448	1.135	69
Extant bird	Charadriiformes	Modern	*Larus*	*marinus*	1.413	1.123	4
Extant bird	Charadriiformes	Modern	*Larus*	*ridibundus*	1.429	1.135	65
Extant bird	Charadriiformes	Modern	*Larus*	*glaucoides*	1.355	1.170	66
Extant bird	Charadriiformes	Modern	*Sterna*	*paradisaea*	1.315	1.126	66
Extant bird	Charadriiformes	Modern	*Sterna*	*forsteri*	1.483	1.144	66
Extant bird	Charadriiformes	Modern	*Chlidonias*	*hybridus*	1.275	1.153	65
Extant bird	Charadriiformes	Modern	*Cepphus*	*grille*	1.523	1.173	66
Extant bird	Charadriiformes	Modern	*Uria*	*lomvia*	1.708	1.252	67
Extant bird	Charadriiformes	Modern	*Uria*	*aalge*	1.598	1.244	64
Extant bird	Charadriiformes	Modern	*Synthliboramphus*	*hypoleucus*	1.528	1.099	66
Extant bird	Colombiformes	Modern	*Zenaida*	*macroura*	1.376	1.031	66
Extant bird	Colombiformes	Modern	*Streptopelia*	*turtur*	1.188	0.948	65
Extant bird	Cuculiformes	Modern	*Cuculus*	*canorus*	1.345	1.116	65
Extant bird	Strigiformes	Modern	*Bubo*	*bubo*	1.286	1.089	65
Extant bird	Strigiformes	Modern	*Tyto*	*alba*	1.073	0.991	68
Extant bird	Strigiformes	Modern	*Strix*	*aluco*	1.194	1.041	65
Extant bird	Coraciiformes	Modern	*Merops*	*apiaster*	1.148	1.013	65
Extant bird	Passeriformes	Modern	*Eremophila*	*alpestris*	1.409	1.127	66
Extant bird	Passeriformes	Modern	*Galerida*	*cristata*	1.292	1.101	65
Extant bird	Passeriformes	Modern	*Calcarius*	*ornatus*	1.302	1.064	66
Extant bird	Passeriformes	Modern	*Bombycilla*	*cedrorum*	1.375	1.165	66
Extant bird	Passeriformes	Modern	*Cardeulis*	*carduelis*	1.294	1.170	65
Extant bird	Passeriformes	Modern	*Cardeulis*	*chloris*	1.428	1.095	65
Extant bird	Passeriformes	Modern	*Fringella*	*coelebs*	1.147	1.111	65
Extant bird	Passeriformes	Modern	*Parus*	*caeruleus*	1.272	1.156	4
Extant bird	Passeriformes	Modern	*Parus*	*major*	1.296	1.083	4
Extant bird	Passeriformes	Modern	*Dendroica*	*virens*	1.333	1.110	66
Extant bird	Passeriformes	Modern	*Dendroica*	*discolor*	1.251	1.129	66
Extant bird	Passeriformes	Modern	*Hirundo*	*rustica*	1.498	1.188	65
Extant bird	Passeriformes	Modern	*Riparia*	*riparia*	1.358	1.135	65
Extant bird	Passeriformes	Modern	*Turdus*	*merula*	1.403	1.138	4
Extant bird	Passeriformes	Modern	*Hylocichla*	*mustelina*	1.266	1.110	66
Extant bird	Passeriformes	Modern	*Luscinia*	*megarhynchos*	1.288	1.077	65
Extant bird	Passeriformes	Modern	*Phoenicurus*	*phoenicurus*	1.241	1.070	65

Extant bird	Passeriformes	Modern	*Erithacus*	*rubecula*	1.230	1.047	65
Extant bird	Passeriformes	Modern	*Icteria*	*virens*	1.224	1.087	66
Extant bird	Passeriformes	Modern	*Sturnus*	*vulgaris*	1.456	1.231	65
Extant bird	Passeriformes	Modern	*Troglodytes*	*aedon*	1.286	1.165	66
Extant bird	Passeriformes	Modern	*Calamospizza*	*melanocorys*	1.255	1.079	66
Extant bird	Passeriformes	Modern	*Passer*	*domesticus*	1.430	1.202	66
Extant bird	Passeriformes	Modern	*Corvus*	*brachyrhynchos*	1.517	1.183	70
Extant bird	Passeriformes	Modern	*Corvus*	*corone*	1.555	1.178	65
Extant bird	Passeriformes	Modern	*Corvus*	*frugilegus*	1.343	1.179	65
Extant bird	Passeriformes	Modern	*Garrulus*	*glandarius*	1.451	1.143	65
Extant bird	Passeriformes	Modern	*Pica*	*pica*	1.429	1.080	65
Extant bird	Passeriformes	Modern	*Pheucticus*	*melanocephalus*	1.328	1.167	66
Extant bird	Passeriformes	Modern	*Pheucticus*	*ludoicianus*	1.316	1.094	66

Reference for Image

1 http://blog.arkive.org/wp-content/uploads/2013/03/short-beaked-echidna-egg.jpg
2 Hughes, R.L. and Carrick, F.N. (1978) Reproduction in female monotremes. Austalian Zooloist, 22, 233-254
3 http://thumbs.dreamstime.com/z/snapping-turtle-eggs-chelydra-serpentina-26052896.jpg
4 D.C. Deeming image
5 http://static.panoramio.com/photos/large/6475358.jpg
6 http://news.turtleconservancy.org/wp-content/uploads/2012/05/Chaco_egg_01May2012_arrow-1024x823.jpg
7 http://news.turtleconservancy.org/wp-content/uploads/2011/12/11-JAPONICA-EGGS_25NOV2011-3.jpg
8 http://cdn2.arkive.org/media/DC/DCFFD1FA-184C-4049-AE00-91FBAA98F2B9/Presentation.Large/Egg-of-western-swamp-turtle.jpg
9 http://wiatri.net/Inventory/WiTurtles/Turtles/Images/OrnateTurtleEgg.jpg
10 http://cdn1.arkive.org/media/41/4119A68B-C614-410C-9DF8-D3EEB8869240/Presentation.Large/Leatherback-turtle-egg-held-in-hand-to-show-size.jpg
11 http://media.cmgdigital.com/shared/img/photos/2012/03/31/70/10/turtle_egg2_499066a.jpg
12 Azevedo et al. (2000)
13 http://dfwurbanwildlife.com/wp-content/uploads/2011/07/mediterraneangecko-eggs-006.jpg
14 David Booth, peronal communication
15 http://i111.photobucket.com/albums/n127/MatthewandKathrynSchaefer/Phelsuma%20borbonica/IMG_3799.jpg
16 http://www.sareptiles.co.za/gallery/albums/userpics/11698/IMG_1616.jpg
17 http://cdn1.arkive.org/media/2D/2D4C9048-1F21-4871-965C-D3AF13218B94/Presentation.Large/Schreibers-green-lizard-egg.jpg
18 http://img.photobucket.com/albums/v510/phillipblack/DSC01062.jpg
19 http://sydneyserpents.blogspot.co.uk/
20 http://www.cccorns.com/forum/attachment.php?s=ed2f2ac1089b322746fe5c9770d7f74b&attachmentid=29728&d=1266796291
21 http://www.naij.com/21981.html
22 http://iansvivarium.com/viewtopic.php?f=4&t=7261
23 Shine, R. (1991) Australian Snakes: A Natural History. Reed Books Pty Ltd., New South Wales

24 J.C. Lü, D. M. Unwin, D. C. Deeming, X. Jin, Y. Liu & Q. Ji (2011).An egg-adult association and its implications for pterosaur reproductive biology. *Science* , 331, 321-324.
25 Wang et al., Sexually Dimorphic Tridimensionally Preserved Pterosaurs and Their Eggs from China, Current Biology (2014), http://dx.doi.org/10.1016/j.cub.2014.04.054
26 Ji, Q., Ji, S.A., Cheng, Y.N., You, H.L., Lu¨ , J.C., Liu, Y.Q., and Yuan, C.X. (2004). Palaeontology: pterosaur egg with a leathery shell. Nature 432, 572.
27 http://www5.city.kyoto.jp/zoo/lang/en/institution/siryositu/digital/egg
28 http://australian-creation.weebly.com/3.html
29 http://www.flickr.com/photos/nationalzoo/7604592046/sizes/k/in/photostream/
30 Mumcuoglu, K. Y. (2012) Mortality of Nile Crocodile (Crocodylus niloticus) Eggs Caused by the Flour Beetle (Tribolium castaneum). Open Journal of Veterinary Medicine Vol. 2 No. 1 (2012) , Article ID: 18113 , 4 pagesDOI:10.4236/ojvm.2012.21002
31 http://crocodilian.com/cnhc/images/!tsch6.jpg
32 http://newswatch.nationalgeographic.com/files/2013/07/DSCN1093.jpg
33 Rogers, J.V. (2000) A complete crocodiloid egg from the lower Cretaceous (Albian) Glen Rose formation, Central Texas. Journal of Vertebrate Paleontology, 20, 780-783.
34 Hirsch, K.F & Kohring, R. (1992) Crocodilian Eggs from the Middle Eocene Bridger Formation, Wyoming. Journal of Vertebrate Paleontology, 12, 59-65.
35 Salgado, L., Coria, R.A., Magalhaes Ribeiro, C.M., Garrido, A., Rogers, R., Simón, M.E. , Arcucci, A.B., Curry Rogers, K., Carabajal, A.P., Apesteguía, S., Fernández, M., García, R.A. and Talevi, M. (2007) Upper Cretaceous dinosaur nesting sites of Rı´o Negro (Salitral Ojo de Agua and Salinas de Trapalco´-Salitral de Santa Rosa), northern Patagonia, Argentina. Cretaceous Research 28 (2007) 392-404.
36 http://web.utk.edu/~museum/archives/dinosaur/images/ha-sauro.jpg
37 *http://www.smithsonianmag.com/science-nature/treasure-trove-of-dinosaur-eggs-found-in-india-54407637/*
38 Schaff, R.J. (2012). Incubation of Contunuoolithus canadensis eggs from the Late Cretaceous Two Medicine Formation of Montana. Master of Science thesis, Montana State University, Bozeman, Montana
39 http://www.visualphotos.com/image/1x3744901/dinosaur_eggs_dinosaur_eggs_china
40 Mikhailov, K.E. (1997) Fossil and recent eggshell in amniotic vertebrates: fine structure, comaprative morphology and classification. Special Papers in Palaeontology, 56, 1-80.
41 JC Lu, peronal communication
42 Kellner, A.W. A. and Campos, D.A. (2000) Brief Review of Dinosaur Studies and Perspectives in Brazil. An. Acad. Bras. Ciênc. vol.72 n.4 Rio de Janeiro Dec. 2000 http://dx.doi.org/10.1590/S0001-37652000000400005
43 López-Martínez, N. and Vicens, E. (2012) A new peculiar dinosaur egg, Sankofa pyrenaica Oogen. Nov. Oosp. Nov. from the Upper Cretaceous Coastal Deposits of the Aren Formation, couth-central Pyrenees, Lleida, Catalonia, Spain. Palaeontology, Vol. 55, Part 2, 2012, pp. 325–339
44 David J. Varricchio , Frankie D. Jackson , Robert A. Jackson , and Darla K. Zelenitsky (2013) Porosity and water vapor conductance of two Troodon formosus eggs: an assessment of incubation strategy in a maniraptoran dinosaur. Paleobiology, 39(2):278-296. 2013.
45 Schweitzer, M.H., Jackson, F.D., Chiappe, L.M., Schmitt, J.G., Calvo, J.O. and Rubilar, D.E. (2002). Late Cretaceous avian eggs with embryos from Argentina. Journal of Vertebrate Paleontology, 22, 191–5.
46 M.H. Schweitzer (pers. Comm, 2013)
47 Ferna´ndez MS, Garcı´a RA, Fiorelli L, Scolaro A, Salvador RB, et al. (2013) A Large Accumulation of Avian Eggs from the Late Cretaceous of Patagonia (Argentina) Reveals a Novel Nesting Strategy in Mesozoic Birds. PLoS ONE 8(4): e61030.
48 Varricchio, D.J. and Barta, D.E. (2014). Revisiting Sabath's "Larger Avian Eggs" from the Gobi Cretaceous. Acta Palaeontolgica Polonica, in press.
49 Kurochkin, E.N., Chatterjee, S. and Mikhailov, K. E. (2013). An embryonic enantiornithine bird and associated eggs from the Cretaceous of Mongolia.

Paleontological Journal, 47, 1252–69.
51 https://fossilpenguins.wordpress.com/tag/eggs/
52 Olson, S.L. and Hearty, P.J. (2013). Fossilized egg indicates probable breeding of Brown Pelican (Pelecanus occidentalis) on Bermuda in the Middle Pleistocene. Proceedings of the Biological Society of Washhington, 126, 169-77.
53 Harrison, T. (2005). Fossil bird eggs from the Pliocene of Laetoli, Tanzania: Their taxonomic and paleoecological relationships. Journal of African Earth Sciences, 41, 289–302.
54 Chandler, R.M. and Wall, W.P. (2001). The first record of bird eggs from the early Oligocene of North America. National Park Service /Natural Resources Geologic Resources Division/Geologic Resources Division Technical Report 01/01. http://www.nature.nps.gov/geology/paleontology/ pub/fossil_conference_6 /chandler.htm.
55 http://www.sciencephoto.com/image/429569/350wm/C0108594-Great_auk_egg-SPL.jpg
56 http://www.sodahead.com/fun/elephant-bird-egg-auctioned-off-for-101813-do-you-have-any-elephant-birds-in-your-area/question-3664641/?link=ibaf&q=&esrc=s
57 Gill, B. J. (2006). A catalogue of moa eggs (Aves: Dinornithiformes). *Records of the Auckland Museum, 43* , 55-80
58 http://emufarming.org/yahoo_site_admin/assets/images/Large_emu_egg_prod.126124407.JPG
59 http://cdn2.arkive.org/media/B2/B2BF2B9D-CCA5-4ED7-83BE-C38847710436/Presentation.Small/Little-spotted-kiwi-egg.jpg
60 http://www.nzmuseums.co.nz/account/3359/object/94184/Brown_Kiwi_Egg_Specimen_Apteryx_mantelli
61 http://i.dailymail.co.uk/i/pix/2012/11/26/article-2238548-1637D015000005DC-104_964x567.jpg
62 http://img2.wikia.nocookie.net/__cb20120623094031/animals/images/1/13/King_Penguin_Egg.jpg
63 http://upload.wikimedia.org/wikipedia/commons/6/61/Manchot_Ad%C3%A9lie_MHNT.jpg
64 http://www.royalalbertamuseum.ca/onlineExhibit/eggs/_images/_egg/gpenge.jpg
65 http://www.egretta.org/galleryeggs.html
66 http://natural-history.uoregon.edu/collections/web-galleries/birds-eggs-image-index
67 Tim Birkhead, personal communication
68 https://www.skullsunlimited.com/products/4578/barn-owl-egg-42mm.htm
69 http://www.illustratedwildlife.com/illustrations/index.php?image_id=760&category_id=3
70 http://upload.wikimedia.org/wikipedia/commons/f/f1/The_Childrens_Museum_of_Indianapolis_-_American_crow_egg.jpg
71 http://www.pinterest.com/pin/427982770810976760/
